# Saffron nephroprotective effects against medications and toxins: A review of preclinical data

**DOI:** 10.22038/IJBMS.2022.61344.13570

**Published:** 2022-04

**Authors:** Batool Zarei, Sepideh Elyasi

**Affiliations:** 1 Department of Clinical Pharmacy, School of Pharmacy, Mashhad University of Medical Sciences, Mashhad, Iran

**Keywords:** Acute kidney injury, Crocin, Crocus, Safranal, Saffron

## Abstract

Toxin and drug-induced nephrotoxicity (DIN) account for about 25% of all acute kidney injury cases and are associated with morbidity and increased utilization of healthcare services. No approved preventive compound is available for DIN. Saffron (Crocus sativus) has important biological properties like antioxidant and anti-inflammatory effects. The protective effects of saffron and its main constituents in different tissues including the brain, heart, liver, kidney, and lung have been confirmed against some toxic materials or drugs in animal studies. This review covers all aspects of saffron’s preventive and therapeutic effects against toxins and DIN including proposed mechanism of action, dosing schedule, and effects on renal biomarkers and histological changes. PubMed, Embase, Scopus, and Web of Science databases were searched by these search terms: “saffron” OR “Crocus sativus” OR “crocetin” OR “crocin “OR “safranal” AND “Drug induced nephrotoxicity” OR “Renal Injury” OR “Kidney Injury” OR “Nephrotoxicity”. All 25 relevant *in vitro* and *in vivo *studies up to the date of publication were included. Promising protective effects were reported particularly on aminoglycosides, cisplatin, and ethanol. Saffron and its constituents significantly prevented biochemical and histopathological changes, mediating via antioxidant, anti-apoptosis, and anti-inflammatory effects. Despite success in animal models, no human study is available in this field and further well-designed clinical trials are necessary for better judgment.

## Introduction

**Figure 1:**
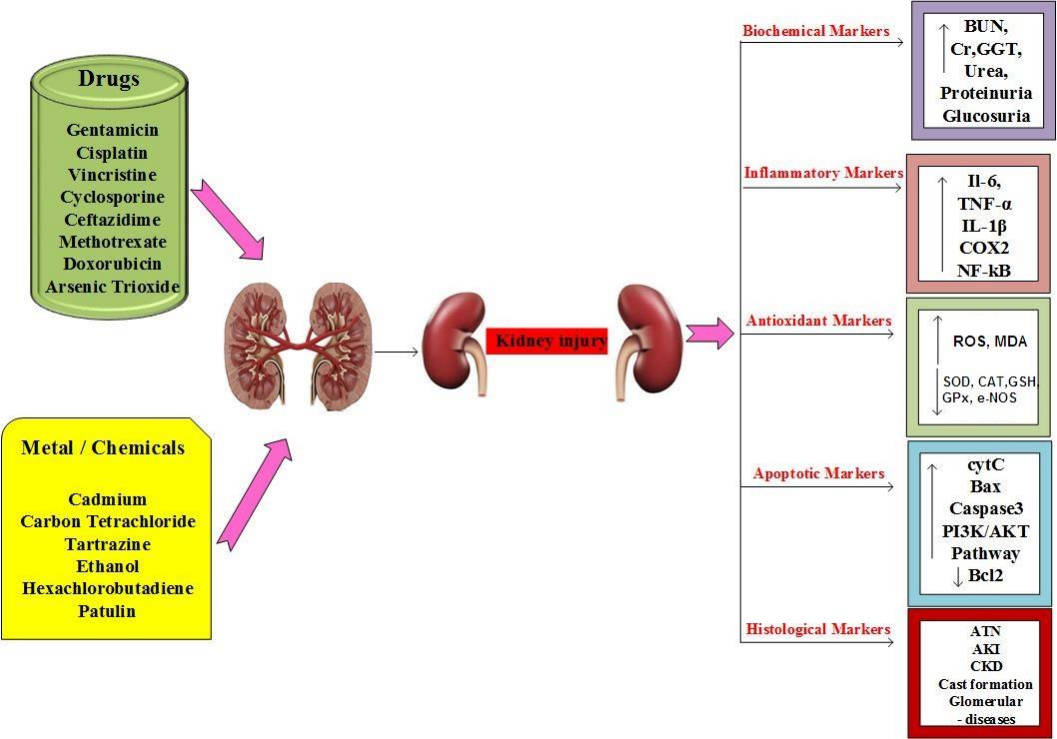
Most probable mechanisms of drug and toxin-induced nephrotoxicity

Due to its unique biochemical, anatomical, and physiological properties, the kidney is a target organ for numerous xenobiotic toxicants, including potentially harmful chemical elements in the environment (1, 2). Contributing factors to its high sensitivity to xenobiotics include abundance of metabolizing enzymes and transporters in the kidney, extremely high renal blood flow*, *and its ability to concentrate different solutes in steps of urine formation (2). Some drugs have potential inherent kidney toxicity, such as aminoglycosides, amphotericin B, and cyclosporine. These effects exert through one or more common pathogenic mechanisms such as thrombotic microangiopathy, glomerular hemodynamic changes, inflammation, crystal nephropathy, rhabdomyolysis, and tubular cell toxicity (3). These effects can largely be due to excessive generation of reactive oxygen species (ROS) that causes damage to cellular macromolecules such as proteins, lipids*, *and DNA*,* ultimately resulting in kidney cell death (4). The most probable mechanisms of drug and toxin-induced nephrotoxicity are summarized in Figure 1. Drug-induced nephrotoxicity (DIN) is likely to be most prevalent among certain patients and in specific medical conditions like elderly, baseline renal failure (glomerular filtration rate (GFR***)*** < 60 ml/min/m^2^*), *volume depletion, concomitant use of nephrotoxins, diabetes, heart failure and sepsis (3). Consequently, available research proves that many medicinal plants can attenuate the biochemical, structural, and functional renal toxicities of a wide spectrum of drugs and toxins representing effective nephroprotective alternatives.

*Crocus sativus* L (commonly known as saffron) is a perennial stemless herb from the Iridaceae family that is cultivated in Iran and a number of other countries including France, Mexico, Greece, India, China, Spain, Turkey, Morocco, Egypt, Azerbaijan, and India (5). Saffron is used as a food additive for enhancing its texture or appearance and preserving flavor (6). The broad spectrum of saffron pharmacologic effects is related to its main constituents including crocetin, crocin, safranal, and picrocrocin (7, 8). Previous studies have evaluated the biological effects of saffron and its constituents including antidepressant and anxiolytic (9, 10), anticonvulsant (11), memory-enhancing (12, 13), antinociceptive (14-16), reducing withdrawal syndrome symptoms (17), improvement of erectile function (18), anticancer (19), antitussive (20), anti-hyperlipidemic, and cardioprotective (21-23) effects which are mostly mediated by anti-inflammatory (14-16), and antioxidant properties (24, 25). Saffron is accepted as a compound with various functions due to its antioxidant effects exerted via direct and indirect mechanisms such as ROS scavenging ability and augmentation of antioxidant responses, respectively (26-28). This effect is confirmed in various *in vitro* and *in vivo* studies (29, 30). Among saffron constituents, crocetin (8,8′-diapocarotene-8,8′-dioic acid) which is a bioactive low molecular weight natural carotenoid compound is mostly responsible for these pharmacological activities. Crocetin increases the reduced intracellular glutathione and enzymes including glutathione (GSH) reductase and glutathione-S-transferase (26). Safranal also possesses the same properties based on some available data (31). Drug/metal/chemical-induced nephrotoxicity prevention is also one of the saffron proposed activities based on available experimental studies and theoretical models (30, 32). Preventing this toxicity would improve patients’ endurance and permit them to use higher dosage for extended periods of time and accordingly enhance the therapeutic impact and increase therapy effectiveness. In this article, we reviewed all available preclinical studies on saffron efficacy as a preventive measure for drug and toxin-induced nephrotoxicity.

## Methods

**Figure 2:**
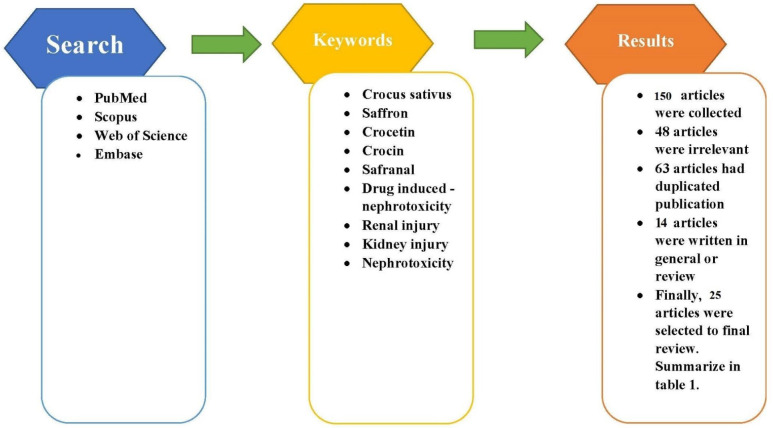
Diagram of the study selection process

In this review article, data were collected by conducting a comprehensive search of electronic databases to find studies on nephroprotective effects of saffron and its active constituents against drugs or toxins. The search was done on PubMed, Embase, Scopus, and Web of Science databases. The search terms included “saffron” OR “Crocus sativus” OR “crocetin” OR “crocin “OR “safranal” AND “Drug induced nephrotoxicity” OR “Renal Injury” OR “Kidney Injury” OR “Nephrotoxicity”. Also, the search process entailed checking reference lists to find additional studies that could help achieve the study’s goal. The inclusion criteria included the availability of online full text or abstract and providing enough information in English, without publication date limit. Criteria for exclusion were: duplicate or unrelated publications. Data collection was carried out between December 2020 and February 2021. Studies were obtained from their inception up to the last of February 2021. The search process and initial selection of eligible studies were performed by the first author. By searching these databases, 150 articles were found. After excluding unrelated (n=48) and duplicated (n=63) articles and also the review or general articles (n=13), eligible articles (case reports/series) were reviewed. No articles were excluded for full-text inaccessibility, or not being available in English. Finally, a total of 25 relevant *in vitro* (n=3) and* in vivo* (n=22) studies up to the date of preparation (April 14, 2021), were included for review (Figure 2). All related articles are reviewed and summarized in Table 1. 

## Results

***Drugs***

*Vincristine*

Vincristine (VCR) is a potential anticancer drug belonging to the family of vinca alkaloids which can be isolated from the leaves of the *Catharanthus roseus *plant (33). VCR is an M*-*phase of the cell cycle-specific drug with time and concentration-dependent activity that can inhibit cancer cell proliferation (34). In short-term exposure and low concentrations, VCR can cause reversible mitotic arrest, prevent segregation of chromosomes**,** and lead to abnormal microtubule polymerization (35). At higher concentration and long-term exposure, VCR can be related to disruption and total depolymerization of microtubule and ultimately lethal cytotoxicity (36, 37). As a result, VCR is the mainstay of solid tumors and hematologic malignancies treatment, including breast cancer, leukemia, and non-Hodgkin lymphomas (NHL)(38). Despite its powerful anti-tumor activity, it has cytotoxicity effects on healthy cells. Several studies have reported cytotoxic effects of VCR on different types of cells such as pancreatic, hepatic, renal cells, and also lymphocytes (39, 40).

Recent studies have shown that overproduction of ROS and oxidative stress (OS) can be considered one of the main mechanisms of renal injury (41).

Saffron’s protective effect on VCR-induced nephrotoxicity has been studied in an *in vitro* study. In this study different VCR doses (0.25, 0.5 and 0.75 mg/kg) alone or plus saffron (0.5 and 1 mg/kg IP) were used for 8 weeks. They showed that administration of VCR can lead to serious renal damage with remarkable increase in the levels of blood urea nitrogen (BUN), creatinine (Cr), and uric acid in a dose-dependent manner. It also considerably raised the mean level of malondialdehyde (MDA), while the total antioxidant capacity (TAC) value was declined. So, probably, VCR causes severe renal impairment through antioxidant depletion and lipid peroxidation (LPx). Administration of saffron extract, particularly with a dose of 1 mg/kg, inhibited renal damage via its antioxidant effect which is shown by decrement in the mean level of MDA and increment in TAC value (42).

*Gentamycin (GM)*

Gentamycin is an aminoglycoside antibiotic initially discovered in 1963 with bactericidal effect on particularly gram-negative bacteria (43). GM is not metabolized but is eliminated unchanged in the urine by glomerular filtration (44). The serious adverse effects of GM consist of ototoxicity, including vestibular and/or cochlear impairment and nephrotoxicity (45). Actually, nephrotoxicity is the main dose-limiting adverse reaction of GM. The reported incidence of nephrotoxicity in different studies varies extensively due to variations in study design, toxicity definitions, patient population, and concomitant risk factors. A reasonable estimation may be 10–20% (46). It is usually presented by a rise in serum creatinine after five to seven days of therapy. It causes acute tubular necrosis (ATN) in proximal tubules resulting in non-oliguric acute kidney injury (AKI) due to a loss in renal concentrating ability (47). At the ultrastructural level, the earliest lesions are an accumulation of myeloid bodies in the lysosome (48). There have been many investigations in recent years proposing an important role for ROS in GM-induced nephrotoxicity (49).

Strategies for minimizing aminoglycoside nephrotoxicity are a once-daily dosing regimen, limiting the duration of therapy, therapeutic drug monitoring, minimizing concomitant other nephrotoxic use, and proper dose adjustment in patients with underlying renal failure (50). Besides, several agents have been used to prevent aminoglycoside nephrotoxicity. Despite their potential, none of them have been accepted clinically for this purpose. Several antioxidant agents including deferoxamine, methimazole, vitamin E, vitamin C, and selenium have been effective in preventing gentamicin nephrotoxicity (50, 51). Protective effects of saffron* and *its active constituents, crocin & safranal on GM-induced nephrotoxicity was hypothesized and tested by three *in vivo* studies, and based on their findings saffron extract, safranal, and crocin showed protective effects. In Ajami *et al*.’s study, the aqueous saffron extract was given to Wistar rats in daily amounts of 40 or 80 mg/kg PO for 10 days to evaluate protection against GM (80 mg/kg/d IP for five days, starting from day 6). Their results showed that saffron extract can diminish GM-induced nephrotoxicity and retain renal histology and function, by inhibition of GM-induced elevated tissue MDA levels (52). In another study saffron extract with a much lower dose showed a renoprotective effect (0.5 mg/kg/d) against the higher dose of GM (100 mg/kg/d) (52), however in that study saffron was given IP and GM as IM injection for 10 d; so the judgment is difficult. It seems that IP injection of saffron with lower doses is more effective than oral use and probably more bioavailable based on this study and Harcheghani *et al*. (42) report. Increase of hydrogen peroxide and superoxide anion production (52), decreased antioxidant defense power (30), increases in multiple proinflammatory cytokines including intercellular adhesion molecule-1 (ICAM-1) and tumor necrosis factor-alpha (TNF-α) (30), release of iron from renal cortical mitochondria to enhance generation of hydroxyl radical (52), and higher concentration of gamma-glutamyl transpeptidase (GGT) in urine (53) were proposed mechanisms of gentamicin nephrotoxicity suggested in these studies. Findings of these studies indicated that GM caused moderate-to-severe renal multiple histological damages with predominant tubular necrosis expanded to the distal portion of proximal tubules and epithelial cell dissociation with cast formation, loss of brush border in large parts of proximal tubules and tubular obstruction, and leukocytes infiltration into interstitium (30, 52, 53). Saffron and its active constituent*s *such as crocin and safranal were able to reduce gentamicin nephrotoxicity that was often characterized functionally by rising of BUN and Cr serum levels and urinary loss of Cr and incidence of proteinuria (particularly albuminuria) (54, 55). In fact, these compounds showed high free radical scavenging activity, reduced products of LPx including MDA, elevated antioxidant capacity in kidneys that diminished cellular injuries which along with its anti-inflammatory properties, limited leukocytes infiltration and may have attenuated the GFR reducing parameters (30, 31, 52). Moreover, crocin probably has vasodilator effects in the kidneys resulting in increased renal oxygen delivery (renal blood flow) and GFR correction (30). Of course, these effects were dose-dependent, because in the study of Ajami *et al.*, saffron 40 mg/kg/d could not change MDA levels (52). This finding corroborates previous studies that revealed a dose-dependent effect for saffron on reducing serum MDA levels (56, 57).

In conclusion, saffron and its derivatives can be used as a pretreatment or coadministration with nephrotoxic drugs such as GM.

*Cisplatin*

Cisplatin** (**CPT) (cis-diamminedichloroplatinum (II); CDDP) is a platinum-based alkylating compound that was approved for the first time in 1978 and served as the foundation of numerous chemotherapy regimens for a broad*-*spectrum of malignancies including small-cell and non-small cell lung cancer, bladder, testicular, ovarian, cervical, and head and neck cancers, which led to improvement of overall survival and also cure rate (58). It is renally excreted and can accumulate in the renal proximal tubules which are selectively sensitive to CPT, lead to cytoplasmic organelle dysfunction, and cause activation of multiple pathways to apoptotic and cellular injury through enhancement of inflammation and oxidative stress (59). Actually, exposure of tubular cells to CPT activates complex signaling pathways that result in tubular cell injury and death. Inflammatory response and also injury to the renal vasculature result in vasoconstriction, reduced blood flow, ischemic injury, and consequently AKI (60). Other possible mechanisms are summarized in Table 1. The nephrotoxic effect of CPT is cumulative and dose-dependent and usually results in dose reduction or withdrawal (61) and also its use is limited to patients with GFR above 60 ml/kg/min (58). So, the effectiveness of chemotherapy has been often limited by this adverse effect in a considerable percentage of patients.

High peak plasma-free platinum concentrations, previous CPT chemotherapy, concomitant nephrotoxic agents, and history of renal failure are the most important risk factors (62-64).

AKI usually displays with a slow rise in serum Cr after five to seven days of therapy*. *Hypomagnesaemia, salt wasting, thrombotic microangiopathy, Fanconi-like syndrome, and anemia are other clinical symptoms of CPT-induced nephrotoxicity (CIN), which could occur in an acute or chronic manner (65).

Different studies have been performed to determine the role of saffron derivatives in CPT-induced nephrotoxicity prevention. In these *in vivo* studies, renoprotective effect of saffron derivatives consist of safranal (200 mg/kg PO), crocin (100, 200, and 400 mg/kg IP), or crocetin (1 & 2 mg/kg IP) as pre- or post-treatment or in combination with CPT (dose range: 5–7 mg/kg) are investigated (66, 67). They indicated that saffron derivatives dramatically prevented CIN in rats (66-68). CPT administration to rats can induce glucosuria and proteinuria**,** which are correlated with Cr elevation and ureaplasma level. Also, CIN was accompanied by a reduction of total thiol, GSH, and total antioxidant status (TAS) level and increase in MDA in kidney tissue (66-68). CPT through generation of free radicals and ROS, inhibition of antioxidant enzyme activity, binding to the renal base transport system, and the following peroxidation of membrane lipids, may exert its nephrotoxicity (67, 68). Histopathological findings revealed a massive injury in the S3 segment of proximal tubules, interstitial nephritis, and degeneration of the tubular epithelial cells (67, 68). Treatment with saffron derivatives depressed LPx in the kidneys which is measured in terms of MDA (32, 68), helped in replenishing the total thiol pool (68), scavenged free radicals (66-68), stabilized the antioxidant enzyme system (66), restored Cr and urea serum levels and urine glucose and protein exertion rate (66-68), and decreased the CPT induced tubular necrosis (68). These effects were dose dependent (66, 68).

A study showed that pretreatment with safranal provided significant protection against CIN and was more effective than post-treatment (67).

El Daly investigated the preventive effects of cysteine (20 mg/kg) together with vitamin E (2 mg/rat), extract of *C. sativus* stigmas (50 mg/kg IP), and *Nigella sativa* seeds (50 mg/kg) against CIN (3 mg/kg). They found that administration of this mixture could partially neutralize many enzyme changes in the kidney induced by CPT. CIN was diminished when saffron or *N. sativa* were given 30 min prior to cisplatin administration. These results suggested the relatively slower excretion rate of CPT by the kidney and/or the slower progression of CIN in comparison with the other nephrotoxic substances (69). 

In conclusion, saffron showed a protective effect against CPT-induced acute kidney injury particularly by reduction of oxidative stress. This effect was dose-dependent, in the same dose range as studies on other nephrotoxins, and mostly occurs if saffron derivatives were administered before CPT injection. But, before a conclusive statement on the potential benefit of saffron as an adjunct to cisplatin therapy, there is a need for further research such as human well-designed clinical trials*.* 

*Cyclosporine (CYC)*

CYC is an immunosuppressive agent which is used to prevent rejection in solid organ transplantation and treatment of various immune-mediated diseases including active Crohn’s disease, nephrotic syndrome, acute ocular Behçet’s syndrome, endogenous uveitis, psoriasis, and rheumatoid arthritis (70). Calcineurin inhibitors (CNIs) nephrotoxicity is the most common and clinically important complication of CYC use, specifically in kidney transplant recipients. It demonstrates either as AKI, which is generally reversible by dose reduction or as chronic progressive renal disease, which is usually irreversible (71-74). There is a lot of evidence about the role of ROS and decreased antioxidant enzyme activity as one of the major mechanisms of CNIs induced nephrotoxicity. Based on *in vivo* and *in vitro* models, depletion of antioxidant enzymes (i.e., catalase (CAT), Glutathione peroxidase (GPx), Glutathione reductase (GR)) and GSH level which leads to LPx increment as an oxidative stress indicator, are important in CNI (29). Besides, CYC directly impacts renal tubular epithelial cells and leads to promoting epithelial to mesenchymal transition, preventing DNA replication and inducing apoptosis (75). Actually, induction of apoptosis by CYC is associated with oxidative stress, endoplasmic reticulum stress, and autophagy. Also, treatment with CYC enhances production of growth factors (like TGF-β), ROS, and LPx, reduces kidney antioxidant capacity, and promotes strong vasoconstriction of afferent arterioles (75, 76). Renal interstitial fibrosis in CYC nephropathy is correlated with osteopontin and TGF-β expression and macrophage accumulation (77). Histopathologically, nephrotoxicity of CYC is characterized by inflammatory cell influx, tubular atrophy, arteriolopathy, striped tubulointerstitial fibrosis, and increased intrarenal immunogenicity (78).

In the only available study in this field, crocetin-loaded lipid nanoparticles are assessed as a detoxifying agent against CYC induced nephrotoxicity in HEK-293 cells by augmentation of endogenous antioxidant enzymes like SOD and CAT, and maintenance of non-enzymatic GSH homeostasis which may be sufficient to minimize the LPx level (evident from TBARS level). As crocetin clinical pharmacological activities could be reduced due to oxidative degradation by different external factors that promote isomerization of trans-form to inactive cis-form, this novel drug delivery system is considered to be more effective and exhibit higher scavenging activity of free radicals, LPx inhibition, and cytoprotection compared with a reference compound. 

Moreover, crocetin either in native treatment or in NPs might prevent opening of the mitochondrial permeability transition pore (MPTP) by superoxide radical scavenging or through stabilization of the mitochondrial membrane potential, which inhibit ROS release from mitochondria to the cytoplasm. 

Nrf2 is a transcription factor presented in the cytoplasm as an inactive form that plays an essential role in antioxidant-response element-mediated expression of phase II detoxifying and antioxidant enzymes, mainly HO-1. In response to CYC-mediated oxidative stress, the Nrf2 pathway translocates to the nucleus and gets activated. However, pretreatment with crocetin (either in native treatment or NPs), increased the cytosolic Nrf2 level and also decreased the expression of HO-1 protein. It is more prominent in crocetin-loaded NPs treated cells in comparison with native treatment. Moreover, it inhibited mitochondrial membrane potential (MMPs), as the richest source of intracellular ROS in cells (29). Further *in vivo* and human studies on crocetin and other saffron components for prevention of CNI are recommended. 

*Ceftazidime (CEF)*

Cephalosporins are some of the most frequently prescribed classes of antibiotics. They provide broad-spectrum antimicrobial coverage and a relatively low incidence of serious adverse effects. The most common adverse reactions of cephalosporins are hypersensitivity reactions often associated with skin manifestations with occasional systemic symptoms. For instance, glomerulonephritis may be seen in association with hypersensitivity angiitis or serum sickness, and also they may cause allergic interstitial nephritis (79, 80). CEF is a third-generation cephalosporin that is used when gram-negative coverage including pseudomonal antimicrobial activity is needed (79).

Cephalosporins rarely have the potential to induce nephrotoxicity in patients receiving very large doses or concomitant with other nephrotoxins. For example, they may potentiate the renal toxicity of aminoglycosides (81).

Researchers investigated the preventive effects of ethanolic extract of *C. sativus* (IP) against GM (IM) and/or CEF (IM)- induced renal toxicity in albino rats. CEF and GM combination induced more kidney injury than individual drugs, and GM more than CEF. This nephrotoxicity was manifested by weight loss, proteinuria, reduced urine output, rise of Scr, BUN, and erythrocyte sedimentation rate (ESR), and electrolyte changes. It was confirmed by histopathological assessment. Extract of *C. sativus* significantly improved the abovementioned changes (82). As we did not have access to the full text of the article, the administered dose of saffron which was effective is not obvious. For better judgment more comprehensive study in this field is necessary. 

*Methotrexate (MTX)*

MTX, an antifolate agent, is an immunosuppressive and chemotherapeutic agent. It is widely used to treat certain types of cancer, autoimmune diseases, and medical abortion (83, 84). Approximately, more than 90% of MTX is excreted unchanged in the urine by glomerular filtration, tubular secretion, and reabsorption**. **It may occur between 36 hr to even 9 years after its use and could even persist 4 months after MTX discontinuation (85). Three main mechanisms have been proposed for MTX-induced nephrotoxicity including crystal nephropathy, through the intratubular precipitation of MTX and its metabolites, direct pharmacological toxicity against renal tubules by inducing over-generation of reactive oxygen radicals in the kidney, and hyperhomocysteinemia in patients with folate metabolism deficiency (86-91). Nephropathy initially is characterized by an asymptomatic increase in Scr level*, *proceeding to tubular necrosis (89). Many studies have demonstrated that treatment with MTX results in elevated MDA levels and MPO activity and reduced CAT activity, GSH levels, and SOD activity in the blood and kidney (92-94). Recently, it has been reported that inflammatory processes such as abnormal production of inflammatory mediators and neutrophil infiltration, are involved in MTX-induced kidney damage (92).

Only one *in vivo* study in this field is available. Jalili and his team have investigated crocin’s preventive effect against the destructive effect of MTX on kidneys. Evidence suggests that MTX induces kidney cell death through enhancement of tumor necrosis factor-alpha (TNFα) expression, nitric oxide synthase (iNOS) up-regulation, and production of nitric oxide (NO) (92, 95-97). The histopathological investigations in this study showed that MTX (20 mg/kg) caused infiltration of lymphocytes by enlarging Bowman’s capsule space, decreasing the glomerular size, increasing the blood cells, and bleeding in the renal tubules. Concomitant use of crocin (12.5- 50 mg/kg IP) with MTX leads to reduction of Scr and BUN levels, reduction of LPx (decreased MDA) and enhancement of antioxidant capacity (increased FRAP) of renal tissue, and slightly degenerative changes with no evidence of necrosis, which could confirm the protective effects of crocin extract against MTX-induced toxicity (95). In conclusion, crocin treatment can prevent MTX-induced renal damage in rats based on this study’s findings. Human studies to determine the exact mechanism of the protective effect of crocin on MTX-induced nephrotoxicity and the optimum dosage of this compound would be needed.

*Doxorubicin*

Doxorubicin (DXR) is a chemotherapy medication utilized to treat various solid tumors and hematologic malignancies including lymphoma, acute lymphocytic leukemia, breast and bladder cancers, and Kaposi›s sarcoma (98). However, its use is limited due to the toxic effects of DXR in multiple organs such as the kidney, heart, testicles, and also hematologic toxicity (99). DXR causes a disbalance between free oxygen radical production and antioxidants. The disturbance of oxidant*/*antioxidant status which has been revealed with LPx and protein oxidation results in tissue injury (100). Even though the precise mechanism of DXR*-*induced renal toxicity is unclear, studies suggest that the toxicity may have happened through generation of free radicals, iron-dependent oxidative injury of biological macromolecules, membrane LPx, and protein oxidation (101). DXR causes alterations in the kidneys of rats including tubular atrophy and increased glomerular capillary permeability (102).

In an *in vivo* study, Hussain *et al*. reported that IP injections of crocin (100 mg/kg/day) for 3 weeks could reduce the toxic effects of DOX (3.5 mg/kg twice weekly for 3 weeks) on rat kidneys. Increased abundance of renal nuclear factor kappa-light-chain-enhancer of activated B cells (NF-*κ*B) mRNA, iNOS, cyclooxygenase 2 (COX2), TNF*α* expression, and reduction of oxidative stress in the kidneys were proposed mechanisms of DXR-induced renal damage. Interestingly, crocin down-regulated the increase in NF-*κ*B mRNA, which in turn decreased iNOS mRNA as well as COX2 and TNF*α* immunoreactivity in renal tissues (103). This study demonstrated that, as adjuvant therapy for doxorubicin, crocin has renoprotective properties in rats. It should be mentioned that DXR and MTX-induced nephrotoxicity are somewhat delayed in comparison with medications like aminoglycoside or cisplatin and a much longer duration of concomitant use of saffron compounds is necessary for being effective.

*Arsenic trioxide*

Arsenic trioxide (ATO) is a traditional Chinese medicine, that was commonly used to treat many diseases, such as rheumatic diseases, psoriasis, and syphilis, for thousands of years (104). During the recent decades, clinical studies confirmed the efficacy of arsenic trioxide in both newly diagnosed and relapsed acute promyelocytic leukemia (APL3). ATO as a single agent can induce complete remission with minimal myelosuppression (105). Oxidative stress, inflammation, and apoptosis are the principal mechanisms of nephrotoxicity induced by ATO (106). ATN and acute tubulointerstitial nephritis have been reported in patients with severe acute arsenic poisoning (107). No established measure for prevention of ATO nephrotoxicity is available. 

For the first time in a rat model, the protective effect of crocetin against renal injury caused by ATO is investigated. The results of this study revealed that ATO (5 mg/kg IP) induced renal morphological alterations such as glomerular destruction, swollen renal tubular epithelial cells, interstitial fibrosis with inflammatory cell infiltration and atrophy, and necrosis of nephrocytes and consequently elevated serum BUN and Cr level. Compared with the control group, oxidative stress markers (such as ROS, MDA, protein carbonyls (PC), and lipid hydroperoxides (LOOH)) and proinflammatory cytokine parameters (TNF-α, and IL-1β) significantly increased and antioxidant enzyme levels (SOD, CAT, GPx, GSH, and total sulfhydryl groups (TSH)) decreased in the ATO group. Furthermore, ATO caused apoptosis via the PI3K/AKT signaling pathway. Pretreatment with crocetin (25–50 mg/kg IP) dramatically attenuated oxidative stress and inflammation and prevented renal injury caused by ATO which may be related to activation of the PI3K/AKT signaling pathway (108). These positive effects should be further investigated before widespread recommendation.

*Vancomycin*

Vancomycin (VAN) is a glycopeptide antibiotic that is commonly used for treating methicillin-resistant *Staphylococcus aureus* (MRSA) infections (109). Vancomycin-induced nephrotoxicity (VIN) is an important consideration and the high trough concentration of vancomycin is the main risk factor for its occurrence (110). Several strategies have been proposed for the prevention of VIN. Animal studies showed beneficial effects of various antioxidants, such as erdosteine, vitamins E and C, and N-acetylcysteine, but their efficacy is not confirmed in well-designed clinical trials (111). In an *in vivo* study on rats, vancomycin (80 mg/kg, IP) caused a significant rise in Scr, BUN, and renal MDA levels, whereas, SOD activity was decreased, when compared with the control group. But administration of aqueous saffron extract (200 mg/kg/BD, IP), 24 hr before VAN, significantly reversed all the abovementioned items. Substantial histopathological changes like destruction of kidney tubules, interstitial edema, epithelial vacuolization, and epithelial desquamation, were also observed with the VAN group. However, administration of saffron extract resulted in a significant reduction of these alterations (112). Further human studies on this compound for prevention of VIN are necessary for better judgment.

***Metal/Chemicals***

*Ethanol*

Ethanol (ETH, also called alcohol, ethyl alcohol) is extensively accessible as a drink worldwide. It is commonly used in cosmetics and personal care products, such as mouth rinse, hair tonic lotion, aftershave, antiseptics, dishwashing liquid, glass cleaners, and in industry, as a solvent (113). Alcohol affects various organ systems of the human body including the liver, lungs, pancreas, kidneys, and digestive, immune, cardiovascular, and central nervous systems (114). The liver is an important organ that metabolizes ethanol via various enzymatic pathways such as microsomal ethanol oxidizing system (MEOS), cytochrome P450 2E1 (CYP2E1), CAT, ethanol dehydrogenase (ADH), and non-enzymatic pathways, however, the kidneys are also sensitive to the damage induced by alcohol (115, 116). Studies have suggested an imbalance between free radicals and antioxidants and generation of ROS which is caused by ethanol metabolism, and results in molecular and cellular damage (117). Excessive use of alcohol can have serious detrimental effects on the kidneys and result in acid-base and electrolyte disorder, reduction of GFR, elevation in serum levels of BUN and Cr, urinary concentrations of glucose and protein, and renal necrosis (118). The protective effects of saffron and its derivative, crocin, against inflammation, oxidative stress, apoptosis, and histopathological and biochemical changes induced by ethanol to the kidneys were evaluated by three animal studies. The results of these studies showed that ETH (50% v/v– 6 ml/kg/day. BW) caused nephrotoxicity manifesting as an increment in the levels of biochemical (TG, LDL, urea, and Cr levels) and inflammatory (IL-6 and TNF-α) markers, reduction of GSH content and enhancement of MDA levels, disturbance in gene expression and apoptosis. They proved that saffron aqueous extract (40 to 160 mg/kg/d), saffron hydroalcoholic extract (167.5 and 335 mg/kg/day), and crocin (10 to 40 mg/kg/d) could reverse all aforementioned abnormalities in rats’ kidneys through anti-LPx, anti-apoptotic, and anti-oxidant effects in a period of 4 weeks; the saffron derivatives were administered intraperitoneally in these studies (119-121). Also, LD50 of the hydroalcoholic extract of saffron is reported about 670 mg/kg which is much higher than the abovementioned doses (119). These findings showed that the preventive effect of hydroalcoholic extracts of saffron is more than its therapeutic effect and exhibited in a dose-dependent manner (119).

*Cadmium*

Cadmium (Cd)is a toxic element that is obtained as a byproduct of zinc production (122). It is one of the important sources of environmental and industrial pollution, obtained through usage of drinking water and foods, breathing in polluted air or tobacco smoke, or from ingestion of contaminated soil and dust particles (123). Cd can accumulate in different organs consisting of the kidney, liver, testicles**, **and pancreas, and negatively impact the functions of these organs (124). Among them, the kidney is known as the main organ of Cd-induced toxicity. The S1 and S2 segments of the proximal tubules are the main target sites. Various mechanisms have also been identified for Cd nephrotoxicity, consisting of oxidative stress, inflammation, cell apoptosis, and glomerular contraction (125).

Zaree and his team evaluated the preventive and therapeutic effects of saffron aqueous extract (100 mg/kg IP) on Cd chloride exposed mouse kidney (30 μmol/kg IP for 3 d) when administered 3 days before or after Cd, respectively. The results showed that cadmium significantly caused kidney failure in an indirect manner, possibly by increasing the free radicals level in various organs and consequently genotoxicity in the DNA of kidney cells. Administration of saffron extract as a prevention or treatment measure caused a significant reduction in DNA damage through antioxidant effects (126). Therefore, the use of saffron as a suitable dietary supplement to protect industrial workers exposed to Cd could be recommended after conducting well-designed human studies.

*Hexachlorobutadiene*

Hexachlorobutadiene (HCBD) is a colorless liquid at room temperature which is widely used in industry to make rubbers, elastomers, transformers, heat-transfer liquids, fungicides, herbicides, and insecticides (127). HCBD is diffused in the environment and pollutes human water and foods. It causes harmful effects in the body and high levels of ROS, and products of LPx are responsible for this toxicity (128, 129). HCBD is known as a strong nephrotoxic through formation of toxic electrophilic metabolites that result in injury of renal tubular epithelial cells (130).

The only available study on the protective effects of saffron against HCBD induced renal injury is proposed by Boroushaki *et al*. They found that treating rats with safranal at doses of 0.25 and 0.5 ml/kg one hour before HCBD (50 mg/kg IP) injection is able to protect kidneys against its nephrotoxicity. Light microscopic examination of kidney sections showed extensive damage in the straight portion of proximal tubules in HCBD and safranal (0.1 ml/kg) treated groups. Preventive treatment with safranal (0.25 and 0.5 ml/kg IP) could decrease the pathological changes and renal biochemical parameters (such as serum urea, and urine glucose and protein exertion) except Scr. So, it seems that the nephroprotective effect of safranal was dose-dependent. Moreover, no significant difference in MDA concentrations, as an indicator of lipid peroxidation, was found between groups. They recommended that HCBD-induced renal necrosis may not be related to oxidative stress. On the other hand, the protective effect of safranal may not be due to its antioxidant activity. Organic anion transporter (OAT) system transports HCBD to the renal proximal tubular cells. Accordingly, the protective effect of safranal may be mediated through OAT inhibition. In addition, safranal might change the metabolism of HCBD by affecting glutathione S-transferase and/or cysteine conjugate b-lyase activity to inhibit toxic thiol formation (131). Further studies in this field are necessary for better understanding. 

*Patulin*

Patulin (PAT), (4-hydroxy-4H-furo(3,2-c) pyran-2(6H)-one) is one of the most important mycotoxins. PAT frequently contaminates apples and apple products, rotten fruits, moldy feeds, and stored cheese (132). PAT is one of the public health concerns because of its potential mutagenic, immunosuppressive, teratogenic, and carcinogenic properties (133, 134). Available evidence has shown that exposure of humans to PAT is extremely toxic to the liver, kidneys, gastrointestinal tract, and the immune system (135). Animal studies indicated that PAT prompts various histological changes in the kidney tissue such as glomerular hypercellularity and shrinkage, hyperplasia of the epithelial lining, and destruction of capillary walls. Furthermore, PAT leads to the loss of microvilli (apical), mitochondria, and brush border of proximal and distal convoluted tubules as well as interstitial inflammatory cell infiltration into renal tissue. In addition, PAT affects the arrangement of mitochondria and cellular cast formation and causes apical aggregation of organelles and formation of irregular heterochromatin in the nucleus (136). Histological analysis of PAT in kidneys showed atrophy of some renal corpuscles and some degenerated glomeruli. Regions of hemorrhage and extravasations were also detected between the tubules of the cortical area (137). Boussabbeh *et al*. found that administration of PAT with dose of 3.75 mg/kg (IP) caused oxidative damages in kidneys through augmentation of the ROS level and peroxidation of lipids and proteins and reduction of the activity of cellular antioxidants such as CAT, SOD, and GSH. The Pre-treatment of mice with crocin, a single IP dose of 50 to 250 mg/kg 3 hr before the PAT administration, prevented PAT-induced oxidative injury in kidneys. Crocin decreased lipid peroxidation and protein oxidation and also balanced oxidant (or pro-oxidants) and antioxidant status by regulating the antioxidant enzymes in the endogenous system (138).

In an *in vitro* study, pretreatment with crocin (250 µM), as an effective free radical scavenger, could alleviate PAT-induced toxicity in embryonic kidney cells (HEK293) by inhibiting ROS formation, endoplasmic reticulum stress, and apoptosis through decrease in GADD34 and GRP78 expressions and reduction of MDA generation (139).

*Tartrazine*

Tartrazine (T) is a yellow-orange easily soluble powder in water (140). It is often applied in the cosmetics and pharmaceutical industry as well as in food products such as cotton candy, energy drinks, and flavored corn chips (141). The metabolites of T, including aminopyrazolone and sulfanilic acid, could lead to the generation of excessive ROS generation, which could in return cause tissue and organ damages (142). These damages often induce diseases such as cancer and aging, and liver, renal, cardiovascular, neurological, and muscle diseases (143).

Erdemli *et al*. evaluated the protective effect of crocin against T-induced nephrotoxicity in Wistar rats. They reported that administration of T (500 mg/kg PO) increased BUN and Scr levels and oxidative stress biomarkers such as SOD, MDA, CAT, and TOS in the renal tissue while decreasing GSH and TAS levels. Also, different levels of inflammatory cell infiltration and vascular and capillary congestion were seen in the renal peritubular interstitial tissue. Co-administration of crocin (50 mg/kg PO) with T for 21 days demonstrated strong antioxidant properties and was able to shift the antioxidant/oxidant balance in favor of antioxidants in kidney tissue. As a result, crocin administration decreased MDA and TOS levels and significantly increased GSH and TAS levels and may exert a preventive effect against T renal toxicity (144).

*Carbon tetrachloride*

Carbon tetrachloride (CCl4) is a highly toxic chemical compound that is commonly used in the dry-cleaning industry (145). CCl_4_ as a volatile solvent poisons many individuals through occupational and environmental exposures (146). Studies demonstrated that CCl_4_ causes disorders in the kidneys, liver, lungs, and testis as well as in blood via free radical formation. Exposure to this organic solvent causes acute and chronic renal failure. Moreover, case studies confirm that CCl_4_ leads to renal diseases in humans (147). CCl_4_ is converted to trichloromethyl radical (CCl_3_-) by cytochrome P450 2E1 in the liver endoplasmic reticulum. CCl_3_- and trichloromethyl peroxyl radical (CCl_3_O_2_•), are presumed to initiate the process of free radical-mediated lipid peroxidation leading to the accumulation of lipid peroxidation products that cause renal injuries (148). Two available studies have evaluated crocin’s protective effect on CCL_4_–induced nephrotoxicity. The results of these studies showed that administration of CCl_4_ (0.2–0.5 ml/kg PO or IP) in rats increased MDA, TOS, BUN, and Cr levels, and renal levels of TNF-α, IL-6, prostaglandin E2, and active caspases-3; GSH, SOD, CAT, and TAS levels were also decreased. Besides, histological studies showed that CCL_4_ leads to glomerular collapse in kidney sections, narrowing and local occlusion in Bowman’s space in certain glomeruli, inflammatory cell infiltration, and congestion. Coadministration of crocin 100 mg/kg/day PO or IP with CCL_4_ for 15–28 days successfully protected against CCl_4_-induced nephrotoxicity in rats. According to these studies, these positive effects could be mediated through modulation of metabolic enzymes, which may result in the reduction of CCl_4_-induced free radical production and lipid peroxidation manifested by declined MDA content in kidneys, induction of antioxidant enzyme activities, and elevation of reduced glutathione levels, and reduction of PG E2, IL-6, and TNF-α levels in kidney tissue, and inhibition of caspase-3 activity and hence could protect kidney cells from death (144, 149)

**Table 1:** Summary of preclinical studies evaluating saffron as a nephroprotective agent

**Nephrotoxic drug/metal/chemical**	**Presentation/Mechanism of toxicity**	**Type of study**	**Result/Mechanism of protection by saffron**	**Ref.**
VCR	Oxidative stress Sig. ↑ in SCr, BUN, and uric acid levels (dose-dependent) Sig. ↑ of MDA level & ↓ in TAC with a dose of 0.75 mg/kg (dose-dependent)	**In vivo**	↓ level of SCr, BUN, and MDA values, and enhancement in serum TAC content with saffron No sig. effect on uric acid level This effect was notable for rats that received 1 mg/kg plant extract (dose-dependent preventive effect).	[42]
		Male Wistar rat (n=5 for each group) Group A: VCR 0.25 mg/kg Group B: VCR 0.5 mg/kg Group C: VCR 0.75 mg/kg Group D: VCR 0.25 mg/kg + saffron 0.5 mg/kg, group E: VCR 0.5 mg/kg + saffron 0.5 mg/kg, Group F: VCR 0.75 mg/kg + saffron 0.5 mg/kg, Group G: VCR 0.25 mg/kg + saffron 1 mg/kg, Group H: VCR 0.5 mg/kg + saffron 1mg/kg, Group I: VCR 0.75 mg/kg + saffron 1 mg/kg, (All groups for 8 w, IP)		
GM	Oxidative stress Increases pro-inflammatory cytokines Sig. increase in SCr, & BUN Tubular necrosis Loss of brush border in proximal tubules Tubular obstruction Leukocytes infiltration into the interstitium	**In vivo** Wistar albino rat (each group 8 rats) Group 1: saline 1 ml/kg Group 2: GM 80 mg/kg/day Group 3: safranal 0.5 ml/kg +GM 80 mg/kg/day after 1 hr (for 6 d, IP)	Sig. ↑ in BUN, Cr, urinary glucose, and protein in group 2 compared with groups 1 &3. No sig. difference between groups 1 & 3.	[31]
		**In vivo**	Sig. ↑ in SCr and BUN & renal tissue MDA level and decrease in the renal tissue FRAP level in GM group All of them sig. reversed by CRO Glomerular atrophy, cellular desquamation, tubular necrosis and fibrosis, epithelial edema of proximal tubules, perivascular edema, vascular congestion & intra-tubular proteinaceous casts in the GM group, all partially recovered by CRO.	[30]
		Male Wistar rats (each group 8 rats) Saline-saline group: N/S at the same volume as the drugs, Saline-crocin group: 100 mg/kg 1-12 days Saline-GM group: 100 mg/kg, from 6th to 12th day. Crocin-GM group: crocin 100 mg/kg from first to 12th day +GM 100 mg/kg from 6th to 12th day. All IP		
		Male Wistar rats (each group 8 rats) Saline-saline group: N/S at the same volume as the drugs, Saline-CRO group: 100 mg/kg 1-12 days, Saline-GM group: 100 mg/kg, from 6^th^ to 12^th^ day. Crocin -GM group: CRO 100 mg/kg from first to 12^th^ day + GM 100 mg/kg from 6^th^ to 12^th^ day. All IP		
		**In vivo**	Sig. ↓ in urinary GGT, Scr, BUN, and necrosis	[52]
		Male Wistar rats Four groups of rats (n=7 for each group) Group 1: NS 1 mL IM Group 2: GM 100 mg/kg/d (IM) Group 3: Aqueous saffron extract 5 mg/kg/d IP Group 4 GM 100 mg/kg/d (IM) +Aqueous saffron extract 5 mg/kg/d IP All for 10 days		
		**In vivo**	Saffron at 40 mg/kg/d sig. reduced BUN and histological scores. Saffron 80 mg/kg/d sig. reduced BUN, SCr, MDA, and histological injury.	[53]
		Male Wistar rats (n=8 for each group) Group 1: N/S for 5 days Group 2: GM 80 mg/kg/d (IP) for 5 days Group 3: aqueous saffron extract (40 mg/kg/d) PO for 10 days Group 4: aqueous saffron extract (80 mg/kg/d) PO for 10 days, Group 5: saffron extract (40 mg/kg/d) PO for 10 days + 80 mg/kg/d GM (IP) starting from day 6 for 5 days Group 6: aqueous saffron extract (80 mg/kg/d)+ GM		
CPT	Increased glucose and protein exertion in urine Increased Scr and urea level Oxidative stress & free radical production Massive injury in the S3 segment of proximal tubules Interstitial nephritis Degeneration of the tubular epithelial cells increased activity of G6PD ↓phosphorylation to oxidation ratio in the mitochondria, indicating reduced ATP production Inhibition of mitochondrial F1F0-ATPase Cellular toxicity Vasoconstriction in the kidney microvasculature Increases the expression of proinflammatory cytokines Direct inhibition of PPAR-alpha activity in renal epithelial cells Induction of hyperlipidemia and accumulation of triglycerides and NEFAs in kidney tissue	**In vivo**	Inhibited lipid peroxidation Reversed increment of MDA and TOS level Sig. increase in kidney GSH level Ameliorated biochemical indices of nephrotoxicity in both plasma and kidney tissues Pretreatment with safranal being more effective	[67]
		Male Sprague–Dawley rats (n=8 for each group), Control group: N/S Group S: safranal Group CP: a single dose CPT IP Group (CPT+S): A single dose of CPT IP before 5 days of safranal post-treatment, group (S+CPT): A single dose of CPT IP following 5 days of safranal pre-treatment. All groups safranal dose: 200 mg/kg gavage CPT dose: 7 mg/kg IP		
		**In vivo**	Sig. ↓ in BUN, Scr, and urinary glucose and protein conc. No histopathologic damage in crocin-treated groups A sig. and dose-dependent ↓ in MDA conc.	[68]
		Rats (n=6 for each group) Group 1: saline 2 ml/day for 4 days Group 2: a single dose of CPT 5 mg/kg on the first day of the experiment. Groups 3 to 5: CRO (100, 200, and 400 mg/kg, respectively), for 4 days followed by a single dose of CPT 5 mg/kg only on 1 day IP		
		**In vivo**	↓ the lipid peroxidation ↑ the activities of antioxidant enzymes The resumption of BUN, uric acid, and Scr in the normal range	[66]
		In fibrosarcoma bearing animals Crt at doses of 1 mg and 2 mg/kg + CPT of 6 mg/kg		
		**In vivo**	Administration of cysteine and vitamin E, *Crocus sativus*, and *Nigella sativa*. Reduced rise of Scr, BUN, and total serum lipid induced by CPT	[69]
		Adult male albino rats (n=6 for each group) Group 1: CPT 3 mg/kg IP for 5 alternate days Group 2: cysteine 20 mg/kg IP + vitamin E 2 mg s.c. 30 min before CPT 3 mg/kg for 5 days. Group 3: cysteine 20 mg/kg IP + vitamin E 2 mg s.c without CPT Group 4: saffron extracts 50 mg/kg IP 30 min before CPT 3 mg/kg IP for 5 days. Group 5: 50 mg/kg saffron extracts without CPT. Group 6: *N*. *sativa* extract 50 mg/kg IP and after 30 min CPT 3 mg/kg for 5 days. Group 7: only *N.* *sativa* extract 50 mg/kg for 5 days. Group 8: The same volume of NS for the same period.		
CYC	ROS production Decreased the activities of SOD, CAT, and GSH levels Increased level of TBARS	**In vitro**	Enhanced free radical scavenging and cytoprotective ability Nullifying the ROS formation Normalization of HO-1 expression by inhibiting nuclear translocation of Nrf2 Prevented MMPs loss	[150]
		HEK- 293 cells CYC =10 μM Crt-loaded NPs= 0.1, 0.5, and 1 μM		
CEF	Proteinuria and reduced U/O Sig. ↑in BUN, Scr, ESR, kidney weights, and bodyweight loss Serum electrolyte changes. Histopathologic changes in kidney	**In vivo**	Sig. prevention of renal injury caused by CEF and/or GM	[82]
		Albino rat ethanolic extract of *Crocus sativus* (IP) once daily, 30 min. before administration of GM or CEF (IM.) alone or in combination for 10 days.		
MTX	↑ in the levels of thiobarbituric acid reactive substance Increased biochemical marker (Scr and BUN), NO, and FRAP level Decreased MDA Morphologic change in kidney	**In vivo**	Sig. ↓ lipid peroxidation ↑in antioxidant capacity of renal tissue Sig. ↓ in NO for all CRO groups ↓ in renal damage in all CRO groups Improvement in biochemical markers of renal function	[95]
		Male rats (n=6 for each group) Group 1 (normal control): NS equivalent to the amount of other injections. Group 2 (control): MTX 20 mg/kg group 3: CRO 12.5 mg/kg group 4: CRO 25 mg/kg group 5: CRO 50 mg/kg group 6: CRO 12.5 mg/kg + MTX 20 mg/kg group 7: CRO 25 mg/kg + MTX 20 mg/kg group 8: CRO 50 mg/kg + MTX 20 mg/kg All injections IP once a day for 28 days		
DXR	Oxidant /antioxidant imbalance in renal tissue Sig. ↑ in renal INOS mRNA relative expression in the DXR group ↑ in NF-*κ*B, iNOS, COX2, and TNF*α *expression increase in glomerular area in the DXR group vs control group ↓ in proximal convoluted tubule area in the DXR group vs normal control	**In vivo**	Down-regulated the ↑ in NF-κB mRNA, which in turn ↓ iNOS mRNA as well as COX2 and TNFα immunoreactivity in renal tissues Improvement in kidney function	[103]
		Male albino Sprague-Dawley rats (n=6 for each group) Control group: NS CRO control group: 100 mg/kg/d DXR group: 3.5 mg/kg twice weekly CRO + DXR group: CRO 100 mg/kg/d+ DXR 3.5 mg/kg twice weekly All groups IP for 3 weeks		
ATO	Morphological alterations in kidney Increment in serum BUN and Scr Increased ROS, MDA, IL-1β, TNF-α, PC, and LOOH Elevated arsenic concentration levels Reduction in SOD, CAT, GPx, GSH, and TSH levels ATO caused apoptosis by elevating CytC, Bax, and Caspase-3 and inhibiting Bcl-2	**In vivo**	Crt reduced oxidative stress in ATO-induced nephrotoxicity Activation of PI3K/Akt signaling pathway led to inhibition of apoptosis Decrement in IL-1β and TNF-α	[108]
		Male adult Sprague-Dawley (n=10 for each group) control group: NS 10 ml/kg Crt pretreatment group: Crt 50 mg/kg + 0.9% NS 10 ml/kg ATO group: ATO 5 mg/kg+ 0.9% NS 10 ml/kg L-Crt group: Crt 25 mg/kg + ATO 5 mg/kg H-Crt group: Crt 50 mg/kg + ATO 5 mg/kg All groups oral Crt six hours before ATO IP for one week		
VAN	Increasing the levels of biochemicals (BUN & Scr) Sig. ↑ in renal MDA levels Sig. ↓ in SOD activity Considerable histopathological changes (destruction of kidney tubules, interstitial edema, epithelial vacuolization, and epithelial desquamation)	**In vitro**	↓ in SCr, BUN concentration and renal MDA levels Sig. ↑ in the level of renal SOD activity A sig. reduction of histopathologic damages to the kidneys	[112]
		Adult male Wistar rats (8 rats in each group) (i) control (ii) saffron (80 mg/kg, IP) (iii) VAN (200 mg/kg/BD, IP) (iv) VAN plus saffron (24 hr before VAN)		
ETH	Increasing the levels of biochemical (BUN & Scr) and inflammatory biomarkers (IL-6 & TNF-α) in kidneys Decline in GSH content Rise of MDA levels Induction of apoptosis Proteinuria	**In vivo**	Improved kidney histopathological damages ↓ inflammatory biomarkers ↓ in MDA levels and ↑ in GSH content ↓ in both mRNA and protein levels of Bax/Bcl**2 **ratio in the kidney of rats	[120]
		Male Wistar rats (n=6 for each group) Group 1: distilled water orally gavaged Group 2: ETH (5 g/kg – 50% v/v) orally gavaged Groups 3, 4, and 5: Aq. Ext. of *Crocus sativus* (40, 80, and 160 mg/kg) IP plus ETH (5 g/kg – 50% v/v) Groups 6 and 7: Aq. ext. 80 and 160 mg/kg IP, respectively. All groups QD for 4 weeks.		
		**In vivo**	↓ pathological damages in the alcoholic rat ↓ in the increased level of Bax/Bcl-2 ratio in mRNA and protein levels in the kidney Prevention of caspase-8, -9, and -3 increase Stop induction of apoptosis	[121]
		Male Wistar rats (n=6 for each group) Group 1: Distilled water gavage Group 2: ETH (50% v/v – 5 g/kg) orally by gavage Group 3: CRO 10 mg/kg + ETH (50% v/v – 5 g/kg) IP Group 4: CRO 20 mg/kg + ETH (50% v/v – 5 g/kg) IP Group 5: CRO 40 mg/kg + ETH (50% v/v – 5 g/kg) IP Group 6: CRO 20 mg/kg IP Group 7: CRO 40 mg/kg IP All groups QD for 4 weeks		
		**In vivo**	Alleviated pathological damages in the alcoholic rat Diminished the increased level of Bax/Bcl-2 ratio in mRNA and protein levels in the kidney Prevention of caspase-8, -9, and -3 increment Stop induction of apoptosis	[119]
		Male Wistar rats (n=6 for each group) Group 1: Distilled water gavage Group 2: ETH (50% v/v – 5 g/kg) gavage Groups 3, 4, and 5: CRO 10, 20, and 40 mg/kg+ ETH (50% v/v – 5 g/kg) IP Groups 6 and 7: CRO 20 mg/kg and CRO 40 mg/kg IP All groups QD for 4 weeks		
Cd	Oxidative stress in kidney tissue & increased levels of free radicals, resulting in genotoxicity	**In vivo**	Antioxidant effect & Prevention of free radical production Sig. decreased DNA damage and cytotoxicity in both pre- and post-treatment animals with Aq. extract of saffron	[126]
		Swiss-Webster mice kidney (in Cd groups: n=8 & in other groups: n=6) Control group: 200 µl daily NS IP for 6 d Group S: Aq. extract 100 mg/kg IP for 3 d then saline 200 µl for 3 d Group Cd: Cd 30 µmol/kg IP for 3 d then saline 200 µl for 3 d Group S-Cd: Aq. extract 100 mg/kg IP for 3 d then Cd 30 µmol/kg IP for 3 d Group Cd-S: Cd 30 µmol/kg IP for 3 d then extract 100 mg/kg IP for 3 d		
HCBD	Sig.↑ in urinary and blood urea conc. Sig. ↑ in urinary concentration of glucose Extensive damage in the straight portion of proximal tubules Entrance to the renal proximal tubular cells via OAT system.	**In vivo**	Inhibition of the OAT system by safranal No change in MDA conc. By safranal Safranal altered the metabolism of HCBD by affecting glutathione S-transferase and/or cysteine conjugate b-lyase activity to prevent toxic thiol formation	[131]
		Wistar albino rats (n=6 for each group) Group 1: corn oil 1 ml/kg Group 2: HCBD 50 mg/kg Groups 3,4,5: safranal 0.5, 0.25, and 0.1 mg/kg + HCBD 50 mg/kg one hour later		
PAT	Oxidative damages in kidneys by increasing free radical generation Increase in lipid and protein oxidation Overexpression of HSP70 in kidneys Decrease in the GSH/GSSG ratio Increased catalase activity Protein carbonyl group formation Cytotoxic Effect Induction of apoptosis PAT triggered ER stress	**In vivo**	Inhibition of PAT-induced glutathione depletion & restoration of inhibited SOD activity ↑ catalase activity & lipid peroxidation Protection of kidney from protein carbonyl group formation	[138]
		Balb C female mice (n=6 for each group) Group 1: 0.1% DMSO in saline (5 ml/kg). Group 2: CRO (250 mg/kg) 3 hr before 0.1% DMSO (5 ml/kg) Group 3: PAT (3.75 mg/kg) Group 4: CRO (50 mg/kg) 3 hr before PAT (3.75 mg/kg) Group 5: CRO (100 mg/kg) 3 hr before PAT (3.75 mg/kg) Group 6: CRO (250 mg/kg) 3 hr before PAT (3.75 mg/kg)		
		**In vitro**	Protection of cells from PAT-induced DNA fragmentation & mortality Reduction of apoptosis Attenuation of ER Stress Decreased oxidative damages	[139]
		Embryonic kidney cells (HEK293) PAT: 15 μM CRO: 250 μM		
T	Sig. ↑ in BUN & Scr Oxidative stress Sig, ↑ in MDA, TOS, SOD & CAT and ↓ in GSH, & TAS Different degrees of extensive collapse in kidney section glomeruli Inflammatory cell infiltration Vascular and capillary congestion in peritubular interstitial tissues Eosinophilic material and degenerated cell debris in the lumen of tubules	**In vivo**	Strong antioxidant properties Sig. ↑ in GSH &TAS in rat kidney tissues and ↓ MDA and TOS levels to the level of the control group Minimal histopathological damage in CRO+T group Lower total damage score than T group	[144]
		Four groups of rats (n=10 for each group) Group C: NS Group CRO: 50 mg/kg/day Group T: 500 mg/kg Group CRO +T: 50 mg/kg CRO +500 mg/kg T All groups for 21 days gavage		
CCl_4_	Increased ratio of kidney weight to 100 g body weight Mononuclear cellular infiltrations in glomeruli Vascular congestion, focal damage, and severe distortion of renal corpuscles with obliteration of the filtration spaces and narrowing of the Bowman’s space in certain glomeruli and occlusion Sig. ↑ in CYP2E1 activity with concomitant ↓ in GST activity Oxidative stress & production of trichloromethyl free radical (CCl3) Sig. ↑ in PGE2, active caspase-3 content, and renal levels of IL-6 and TNF-*α*	**In vivo** Male Sprague–Dawley rats (n=10 for each group), Group 1: Sterile corn oil in a dose of 0.2 ml/ 100 g, two consecutive days/ week starting from day 4. Group 2: CCl_4_ 0.2 ml/100 g for two consecutive days/ week starting from day 4 Group 3: CRO, 100 mg/kg starting from day 1. Group 4: CRO + CCl_4_ All for 3-week IP	Inhibition of lipid peroxidation & induction of antioxidant enzyme activities ↑ of reduced glutathione level via induction of genes transcriptions Inhibition of caspase-3 activity Inhibition of inflammation by abrogation of PGE2, IL-6, and TNF-α levels in kidney tissue	[149]
		**In vivo**	Sig. ↓ in MDA, TOS, BUN & Scr levels Sig. improvement in glomerular & tubular damage ↑ in GSH levels and ↓ in MDA levels in the kidney tissue	[151]
		Wistar rat (n=10 animals each group) Group 1: NS 1 ml/kg/day Group 2: corn oil 1 ml/kg/day Group 3 :100 mg/kg/day CRO Group 4: CCl_4_ 0.5 ml/kg every other d. Group 5: CRO 100 mg/kg/day + CCl4 0.5 ml/kg every other day. All for 15 d orally (via gavage)		

N.S: Normal saline; BUN: Blood urea nitrogen; CAT: Catalase; GSH: Glutathione; GPx: Glutathione peroxidase; LPO: Lipid peroxidation; ROS: Reactive oxygen species; SOD: Superoxide dismutase; MDA: Malondialdehyde; FRAP: ferric reducing ability of plasma; TNF-α: Tumor necrosis factor-α; GGT: Gamma-glutamyl transpeptidase; NEFAs: Nonesterified fatty acids; PPAR: Peroxisome proliferator-activated receptor –alpha; PC: Protein carbonyls; LOOH: lipid hydroperoxides; TSH: Total sulfhydryl groups; ER: endoplasmic reticulum; GM: Gentamycin; CPT: Cisplatin; VCR: Vincristine; CYC: Cyclosporine; CEF: Ceftazidime; MTX: Methotrexate; DXR: Doxorubicin; ATO: Arsenic trioxide; Cd: Cadmium; CCl_4_: Carbon tetrachloride; T: Tartrazine; ETH: Ethanol; HCBD: Hexachlorobutadiene; OAT: organic anion transporter; GSSG: oxidized glutathione; CRO: Crocin; Crt: Crocetin; IP: Intraperitoneal; IM: Intramuscular; FRAP: ferric reducing/antioxidant power

**Figure 3:**
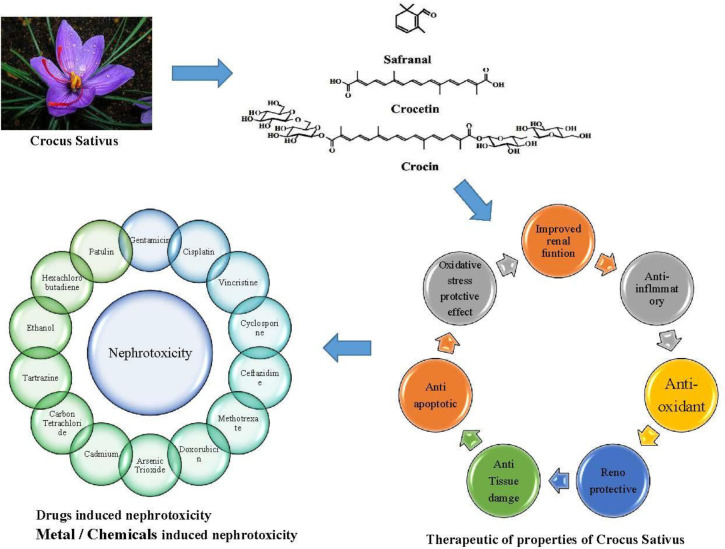
Most proposed mechanisms of saffron induced nephroprotection

## Conclusion

Drug-induced acute kidney injury is one of the major causes of AKI. In recent years, there is a growing number of hospitalized patients with toxin or drug-induced renal failure. Despite numerous supportive recommendations including avoiding dehydration and concomitant nephrotoxic medication use, suitable electrolyte replacement, and dose adjustment based on kidney function, about 10% to 30% of treated patients experience nephrotoxicity. The medicinal plants, by presence of bioactive compounds, play an important role in prevention of medication adverse reactions including nephrotoxicity. Saffron is accepted as an antioxidant compound that exerts its effects via direct and indirect mechanisms such as ROS scavenging ability and augmentation of antioxidant responses, respectively. In this review, all *in vivo* and *in vitro* studies are summarized to conclude the efficacy of saffron and its active constituents in protection against DIN. All 25 reviewed studies reported the nephroprotective effect of saffron and its constituents (eight studies on aqueous or ethanolic extract of saffron, 3 studies on safranal, 11 studies on crocin, and 3 studies on crocetin) against nephrotoxic drugs and toxins. Biochemical improvement of kidney function in comparison with the control group was obvious in all 25 studies and histopathological promising effects were shown in 10 studies. Besides attenuation of ROS production, proposed by a significant reduction of MDA, LPx, and TOS and increment of TAC, GSH, and SOD which was the main defined mechanism for prevention of nephrotoxicity by saffron (presented in 18 studies), anti-apoptosis presented by inhibition of PI3K/AKT pathway and BAX/Bcl2 ratio and caspase-3, 8, and 9 reduction (in 6 studies) and anti-inflammatory effect by reduction of TNF-α, IL-6, COX-2, NO and HO-1 expression (presented in 4 studies) are involved in saffron preventive effects. It is worth mentioning that in just one study on HCBD, the authors recommended that HCBD-induced renal necrosis may not be related to oxidative stress, and safranal protective effect may not be due to its antioxidant activity. The most mentioned mechanisms of saffron and its constituents’ nephroprotective effects in studies are summarized in Figure 3. It has also been revealed that saffron was more effective when it was administered prior to the nephrotoxic drug or toxin. In most studies, saffron was administered intraperitoneally, but its oral use was also assessed in some studies and was effective. The best effective dose and duration of saffron derivative use cannot be judged as a limited number of studies on each saffron constituent were done against each nephrotoxic agent. However, no human study is performed until now in this field, and for better judgment well-designed clinical trials are necessary. Also, some other possible limitations restrict the use of this plant in humans including concerns about administration of high dose saffron in humans by converting animal dose and possibility of pharmacokinetic and pharmacodynamics interaction between saffron and patients’ other drugs. 

## Authors’ Contributions

SE Provided study conception and design; BZ Performed data analysis and draft manuscript preparation; SE Critically revised the paper and supervised the research; SE and BZ Approved the final version to be published.

## Data Availability

 Data can be obtained from the authors upon request.

## Financial Support

This work has been supported by Research Affairs of Mashhad University of Medical Sciences, Mashhad, Iran. 

## Conflicts of Interest

The authors declare that they have no known competing financial interests or personal relationships that could have appeared to influence the work reported in this paper. 
